# The Dual Orexin/Hypocretin Receptor Antagonist, Almorexant, in the Ventral Tegmental Area Attenuates Ethanol Self-Administration

**DOI:** 10.1371/journal.pone.0044726

**Published:** 2012-09-21

**Authors:** Subhashini Srinivasan, Jeffrey A. Simms, Carsten K. Nielsen, Steven P. Lieske, Jade J. Bito-Onon, Henry Yi, Frederic Woodward Hopf, Antonello Bonci, Selena E. Bartlett

**Affiliations:** 1 Ernest Gallo Clinic and Research Center, University of California San Francisco, Emeryville, California, United States of America; 2 Translational Research Institute, Queensland University of Technology, Brisbane, Queensland, Australia; 3 Intramural Research Program, National Institute of Drug Abuse, Baltimore, Maryland, United States of America; 4 Department of Neurology, University of California San Francisco, California, United States of America; 5 Solomon Snyder Department of Neuroscience, John Hopkins University, Baltimore, Maryland, United States of America; 6 Department of Mental Health, San Francisco Veteran Affairs Medical Center, San Francisco, California, United States of America; McLean Hospital/Harvard Medical School, United States of America

## Abstract

Recent studies have implicated the hypocretin/orexinergic system in reward-seeking behavior. Almorexant, a dual orexin/hypocretin R_1_ and R_2_ receptor antagonist, has proven effective in preclinical studies in promoting sleep in animal models and was in Phase III clinical trials for sleep disorders. The present study combines behavioral assays with *in vitro* biochemical and electrophysiological techniques to elucidate the role of almorexant in ethanol and sucrose intake. Using an operant self-administration paradigm, we demonstrate that systemic administration of almorexant decreased operant self-administration of both 20% ethanol and 5% sucrose. We further demonstrate that intra-ventral tegmental area (VTA) infusions, but not intra-substantia nigra infusions, of almorexant reduced ethanol self-administration. Extracellular recordings performed in VTA neurons revealed that orexin-A increased firing and this enhancement of firing was blocked by almorexant. The results demonstrate that orexin/hypocretin receptors in distinct brain regions regulate ethanol and sucrose mediated behaviors.

## Introduction

The hypocretin/orexinergic system plays an important role in various neurobiological processes involved in maintaining homeostatic mechanisms in the body. Orexin (hypocretin) neuropeptides A and B are produced in neurons of the lateral, perifornical nucleus and dorsomedial hypothalamus [1], [2], and target two types of orexin/hypocretin receptors. Orexin-A has a greater binding affinity to orexin R_1_ receptor (Ox-R1) than orexin-B, whereas both peptides bind to the orexin R_2_ receptor (Ox-R2) with equal affinity [2]. Deficiencies in the genes either encoding orexin or the Ox-R2 receptor result in narcolepsy, providing evidence that orexin peptides are involved in the regulation of sleep and wakefulness [3], [4]. Multiple brain regions receive orexinergic fiber input from the lateral hypothalamus (LH) and perifornical area [1], [5], [6]. The ventromedial hypothalamic nucleus, the arcuate nucleus and the paraventricular nucleus of the hypothalamus all receive orexinergic input and are involved in food intake. Intracerebroventricular (ICV) injections of orexin-A and orexin-B increase food intake [2], while an orexin-A antibody and orexin receptor antagonists reduce food intake, suggesting that orexin peptides play an important role in energy homeostasis [7], [8]. In addition, the locus coeruleus, the nucleus of the solitary tract, rostral ventrolateral medulla and the lateral paragigantocellular nucleus are involved in blood pressure regulation and receive dense orexinergic input [5]. Increases in blood pressure and heart rate have been observed upon ICV injection of orexin confirming the involvement of orexins in autonomic responses [9].

In addition to homeostatic functions, orexins play an important role in drug-seeking behaviors. The initial link between orexins and reward-seeking behaviors was first established when it was shown that morphine withdrawal induces orexin gene expression in the neurons of the lateral hypothalamus and orexin receptor knock-out mice display attenuated morphine dependence [10]. Dense orexinergic innervation is observed in the ventral tegmental area (VTA), a region widely implicated in the natural and drug reward circuitry of the brain [5], [6]. Intra-VTA administration of Ox-R1 antagonist, SB-334867, suppresses morphine place preference by direct activation of the mesolimbic dopamine system [11]. Furthermore, *in vivo* administration of SB-334867 blocks locomotor sensitization to cocaine and occludes cocaine-induced potentiation of excitatory neurotransmission in VTA dopamine neurons [12].

Although many studies demonstrate the role of orexin receptors in cocaine- and morphine-mediated behaviors [11], [12], [13], the role of the orexin receptors in alcohol-mediated behaviors has been less thoroughly examined. We previously demonstrated that systemic administration of SB-334867 decreased operant self-administration of ethanol and yohimbine-induced reinstatement of both ethanol and sucrose-seeking in rats [14]. In addition, SB-334867 reduces ethanol consumption and cue-induced reinstatement of alcohol-seeking after prolonged abstinence in ethanol preferring rat models [15], [16], [17]. The selective Ox-R2 receptor antagonist, JNJ-10397049, has recently been shown to attenuate ethanol-mediated behaviors [18]. However, it is yet to be determined if orexin enhances ethanol- and sucrose-seeking behavior through action within the VTA.

Excessive alcohol intake, dependence and abuse are serious health conditions affecting the physiological and emotional health of millions of individuals. Despite the need for effective therapeutics for the treatment of this disorder, very few medications exist to provide relief. Three medications have been approved by the U.S. Food and Drug Administration for the treatment of alcoholism: disulfiram (Antabuse™), naltrexone (ReVia™), and acamprosate (Campral™). However, all three suffer from limited efficacy and poor patient compliance [19], [20], [21]. The opioid receptor antagonist, naltrexone, has demonstrated the most consistent effect in reducing alcohol consumption in the context of behavioral therapy, yet these effects are observed in only a relatively small subset of patients with alcohol use disorders (AUDs) [22], [23], [24]. Thus, there remains a critical need for research on alternate targets to better serve the patient population. The orexinergic system presents a novel therapeutic target for preventing relapse to drug and food addiction. Almorexant, a competitive Ox-R1 and Ox-R2 antagonist, was in Phase III clinical trials for sleep disorders [25]. Here we demonstrate the efficacy of almorexant in reducing ethanol and sucrose-seeking, and show that the effect of orexins in ethanol-seeking is mediated, at least in part, by the VTA. This work provides information on the utility of orexin receptor antagonists in the pharmacotherapy of AUDs and maladaptive food consumption.

## Materials and Methods

### 1. Ethics Statement

All procedures were pre-approved by the EGCRC Institutional Animal Care and Use Committee and were in accordance with NIH guidelines for the Humane Care and Use of Laboratory Animals. The use of human cell lines (Biological Use Authorization (41433-BU-01-BNC) was approved by the Office of Environmental Health and Safety of the University of California, San Francisco.

### 2. Subjects

For behavioral experiments, male, Long-Evans rats weighing 150–180 g upon arrival (Harlan, Indianapolis, IN, USA) were individually housed in ventilated Plexiglas cages. For *in vitro* electrophysiological studies, male, Long-Evans rats (Harlan) and Sprague-Dawley rats (Harlan) were received at postnatal day 18 and given a minimum of five days to habituate to the housing conditions before tissue collection. Rats were housed in a climate controlled room on a 12 hour light-dark cycle (lights on at 0700 hours). Food and water were available *ad libitum* in the home cage throughout the experiments.

### 3. Drugs and Chemicals

Almorexant hydrochloride was synthesized by Suzhou Rovathin, Jiangsu, China and dissolved in 2% dimethyl sulfoxide (DMSO) and 25% β-cyclodextrin in saline for systemic injections (i.p.) and 100% DMSO for intra-cranial infusions. The 20% ethanol (v/v) solution was prepared using 95% ethyl alcohol (Gold Shield Chemical Ac., Hayward, CA, USA) and filtered water. Five percent sucrose (Fisher Scientific, NJ, USA) solutions were prepared in filtered water. DMSO, β-cyclodextrin, orexin-A, orexin-B, probenecid and 2-hydroxy-ethylpiperazine-*N*-2-ethane sulphonic acid (HEPES) were purchased from Sigma-Aldrich® (St. Louis, MO, USA) and SB-334867 was purchased from Tocris (Minneapolis, MN, USA). Hygromycin and Hank's Balanced Salt Solution were purchased from Invitrogen (Chicago, IL, USA) and FLUO-3 dyes were purchased from Molecular Devices (Sunnyvale, CA, USA).

### 4. Behavioral experiments

#### A. Self-Administration: Description of the Apparatus

Self-administration training was conducted in standard operant conditioning chambers (Coulbourn Instruments, Allentown, PA, USA) enclosed in sound-attenuating cubicles, and equipped with a fan for ventilation and reduction of background noise. Each operant conditioning chamber consisted of two retractable levers on the right wall (4 cm above the grid floor, 12 cm apart) along with stimulus lights 2 cm above each lever, and a liquid dipper system placed centrally between the two levers. A house light was present on the wall opposite to the levers, which remained on during the operant conditioning sessions. An apparatus that emits a tone under specific conditions was also present. In a fixed ratio paradigm, upon correct (active) lever press(es), the stimulus light above the active lever was illuminated and accompanied by a tone for 3 s to indicate availability of reward in the dipper receptacle. The dipper port was illuminated for 10 s indicating the availability of the dipper cup, which had to be actively licked (as recorded by a lickometer) during the 10 s period to be counted as a reinforcer earned. Otherwise, the cup fell and the event was recorded as a null response. The presence of the lickometer, therefore, provides information about whether the animals were drinking the solution they were responding for, allowing for more accurate estimates of ethanol and sucrose intake (g/kg). The number of reinforcers (availability of 5% sucrose or 20% ethanol in the dipper receptacle) recorded per operant self-administration session was determined by the total number of reinforcers offered minus the null responses. Upon pressing the second inactive lever, no reinforcer, cue light, or auditory stimuli were presented and the event was merely recorded as a measure of non-specific behavior. Stimulus, fluid delivery, and operant responses were all controlled and recorded using the Graphic State 2.0 software (Coulbourn Instruments).

#### B. 20% Ethanol Self-Administration and 5% Sucrose Self-Administration

Using the apparatus described above, daily 20% ethanol self-administration was initiated in three separate groups of Long-Evans rats (n = 9–15 per group) using a previously described method [26]. Importantly, food and water were available *ad libitum* at all times in the home cage throughout the training and no initiation procedures (such as sucrose fading) were employed. On the first day of training, animals were placed in the operant conditioning chambers for a 14-hr overnight session on an Fixed Ratio 1 (FR1) schedule of reinforcement (0.1 ml after a single lever press), with 20% ethanol solution as the reinforcer. These FR1 overnight sessions were performed five days per week for a total of 12 sessions. During these sessions, only the active lever was available for the rat to press to establish lever pressing behavior. Following the completion of these sessions, rats were then exposed to 45-min FR1 sessions for a total of 6 sessions. In the third phase of training, the sessions were reduced to 30 min periods and the work ratio was increased to an FR3 schedule of reinforcement (three active lever presses required for 0.1 ml reinforcer). The inactive lever was also introduced at this time. Rats continued on the FR3 protocol with 20% ethanol as the reinforcer for a minimum of 20 sessions. We have previously shown that animals trained using this procedure consume approximately 1.5 g/kg in a 30 min session, which results in mean blood ethanol concentrations around 60 mg% [26]. Animals not reaching 0.3 g/kg ethanol intake per session were excluded from further study.

In an independent series of experiments, two groups of Long-Evans rats (n = 10–14 per group) were trained to self-administer sucrose using the protocol described above, where 20% ethanol was substituted with 5% sucrose as the reinforcer at all stages of the training. The training was identical to the 20% ethanol self-administration training and included 12 overnight FR1 sessions, six 45 min FR1 sessions, and a minimum of twenty 30 min FR3 sessions. Animals trained to respond for sucrose that failed to reach 30 active lever presses per session were excluded from further study.

#### C. Systemic Almorexant Injection Following Self-Administration Training

After establishment of stable responding for 20% ethanol and 5% sucrose, each group of rats trained to respond for either solutions (n = 14 for ethanol and n = 12 for sucrose) was tested with systemic almorexant to assess the effect of the compound on operant responding and fluid intake. All rats received all four treatment doses (vehicle, 3, 10 and 15 mg/kg; i.p., 30 min prior to the operant session) and each injection was given seven days apart using a Latin square design. Thus, each rat served as its own control.

#### D. Surgery and Intra-VTA and Intra-Substantia Nigra Pars Reticulata (SNr) Almorexant Microinfusions

Two separate groups of rats trained to self-administer 20% ethanol (n = 14 for the VTA and n = 9 for the SNr) and one group trained to self-administer 5% sucrose (n = 9, for the VTA) were continuously anesthetized with isoflurane during surgery. Four holes were drilled for screws, and two other holes were drilled for the placement of the cannulae. Single guide cannulae (C315G, 26 gauge; Plastics One) were bilaterally aimed dorsal to the VTA (n = 14 for ethanol, n = 9 for sucrose; AP −5.3, ML +/−2.05, DV −7.33 at a 10° angle) and SNr (n = 9, ethanol trained animals; AP −5.4, ML +/−3.80, DV −6.91 at a 10° angle) according to Paxinos and Watson [27]. Animals were given a minimum of five days to recover from surgery after which they were returned to self-administration training for a minimum of two weeks before testing, at which time they were habituated to handling and the microinjection procedure. For infusions, almorexant was dissolved in 100% DMSO and infused in a volume of 0.3 µl per side. We have previously shown that infusions of the same volume of 100% DMSO into the central amygdala does not cause cell death [28]. Almorexant or vehicle was infused via a 10 µl Hamilton syringe into the VTA and SNr via injection cannulae extending 1.0 mm beyond the guide cannula tip. Due to the small size of the VTA and SNR, and to limit the possible diffusion, almorexant was microinfused over 2 min. The injectors then remained in position for an additional 1 min. The order of almorexant dose infused was counterbalanced across all subjects. Animals trained to respond for 20% ethanol and 5% sucrose and cannulated in the VTA received all four treatment doses (vehicle, 10, 15 and 30 µg per side; intra-VTA, 10 min prior to the session) and each infusion was given seven days apart using a Latin square design. Thus, each rat served as its own control. As a site specific anatomical control, ethanol-trained animals cannulated in the SNr were infused with the high dose of almorexant and vehicle in a counterbalanced design with seven days between infusions (vehicle and 30 µg per side; intra-SNr, 10 min prior to the session).

#### E. General Locomotor Activity

Locomotor studies were run in activity-monitoring chambers (40×40 cm) with horizontal photo beams (Med Associates, St Albans, VT). Horizontal locomotor activity was monitored at 100 ms throughout the sessions. The study was run in 4 daily 2-hour-sessions as described previously [14]. In brief, after habituation to the boxes (Days 1, 2, and 3) and saline injections (Days 2 and 3), almorexant testing was conducted on Day 4. Data from Day 3 was used to assign naïve animals to one of two treatment groups (vehicle, or almorexant, 15 mg/kg, i.p., n = 6 per group). Following 60 min of habituation to the locomotor chamber on Day 4, a single injection of the assigned treatment was given, after which the session continued for an additional 60 min. Further, this 60 min test period is identical to the test period for the operant self-administration studies, where almorexant was administered systemically (30 min pretreatment +30 min session = 60 min). Data was collected across the entire 2-hour-session and recorded as ambulatory distance and stereotypic counts.

### 5. Generation of orexin receptor cell lines and Fluorescence Based Calcium Assay

Written approval for use of human DNA for the generation of mammalian cell lines, Human Embryonic Kidney Cells (HEK-293) expressing Ox-R1 and Ox-R2 receptors was obtained from the University of California, San Francisco, Office of Environmental Health and Safety. The coding sequences of proteins for Ox-R1 and Ox-R2 receptors were amplified by polymerase chain reaction and subcloned into the pcDNA 3.1 vector. All amplified products were completely sequenced and confirmed. The resulting construct was transfected into HEK-293 cells. Stably transfected clones were selected by flow analysis cytometry sorting. For generation of stable cell lines, single colonies were chosen and propagated in the presence of 200 µg/ml hygromycin. The expression of either orexin receptor in a given cell line was verified using immunohistochemistry followed by confocal miscrosopy. The selected hygromycin resistant cells expressing Ox-R1 and Ox-R2 receptors were plated in 96-well clear bottom black microplates at a density of approximately 40,000 cells/well in DMEM/10% FBS media (100 µl/well) and maintained at 37°C, 7% CO_2_ for 24 hours prior to calcium mobilization assay. On the day of the calcium assay, the cells were loaded with a membrane permeable calcium sensitive fluorophore dye (FLUO-3) diluted in assay buffer (HANKS Balanced Salt Solution supplemented with 20 mM (4-(2-hydroxyethyl)-1-piperazineethanesulfonic acid (HEPES), 2.5 mM probenecid, pH 7.4; 100 µl/well) and incubated at 37°C for 60 min. The calcium mobilization assay was performed using the FlexStation apparatus (Molecular Devices) with the following acquisition settings: excitation = 485 nm; emission = 525 nm at 21°C. Following a pre-assay baseline reading of each well, the intracellular calcium release in HEK-293 cells individually expressing Ox-R1 or Ox-R2 was measured following treatment with orexin-A (at Ox-R1), orexin-B (at Ox-R2). Data was acquired at 1 pt/s for 20 s prior to ligand addition and for 120 s following ligand addition (1 fM-10 µM; 50 µl/well of 5× ligand solutions in assay buffer; each concentration in triplicates). For the inhibition experiments, SB-334867, or almorexant (10 pM-100 µM) was added to the wells (2 µl of 100× ligand solutions in assay buffer added into a total volume of 200 µl per well; each concentration in triplicates) and incubated for 30 min prior to measurement of intracellular calcium release by orexin-A (100 nM) in Ox-R1 or orexin-B (10 nM) in Ox-R2 receptors.

### 6. Electrophysiology

Male Long-Evans rats (p22–30) and male Sprague-Dawley rats (p22–30) were deeply anesthetized with pentobarbital (100 mg/kg, i.p.). Transcardial perfusion was performed with ∼30 ml of ice-cold aCSF (in mM: 75 sucrose, 87 NaCl, 2.5 KCl, 1.25 NaH_2_PO_4_, 7 MgCl_2_, 0.5 CaCl_2_, 25 NaHCO_3_) saturated with 95% O_2_ and 5% CO_2_. The brain was rapidly removed and horizontal midbrain slices (230 µm) were obtained in ice-cold sucrose containing aCSF saturated with 95% O_2_ and 5% CO_2_. Slices were transferred to the recovery chamber containing aCSF (in mM: (126 mM NaCl, 2.5 mM KCl, 1.2 mM NaH_2_PO_4_, 1.2 mM MgCl_2_, 2.4 mM CaCl_2_, 18 mM NaHCO_3_, 11 mM glucose, with pH 7.2–7.4 and mOsm 304–306) and saturated with 95% O_2_ and 5% CO_2_. Slices were incubated at 31°C for at least 1 hr prior to recordings, with 1 mM ascorbic acid added to the aCSF in recovery chamber just before adding the first slice.

Slices containing the VTA were transferred to a recording chamber and superfused with continuously flowing bath solution (∼2 ml/min) of aCSF saturated with 95% O_2_ and 5% CO_2_ (described above) at 31°C. Picrotoxin (50 µM) was added to the aCSF to block GABA-A receptor-mediated inhibitory postsynaptic currents. Neurons were visualized using a microscope with infrared differential interference contrast optics (Olympus, USA). Glass pipettes of 3–5 MΩ resistance in the bath were pulled in Narishige puller (PP-830, Narishige, Japan) and filled with potassium methanesulfonate containing internal solution (containing (in mM): 130 KOH, 105 methanesulfonic acid, 17 HCl, 20 HEPES, 0.2 EGTA, 2.8 NaCl, with 2.5 mg/ml Mg-ATP, 0.25 mg/ml GTP, pH 7.2–7.4, and 278–285 mOsm). Cells in the VTA were identified by the position medial to the medial terminal nucleus (MT) of the accessory optic tract.

Once a giga ohm seal was established in cell-attached configuration, spontaneous firing rates of cells were monitored using the gap-free protocol in Clampex 9.0 acquisition software (Molecular Devices). Only cells which displayed spontaneous firing were included in this study. Voltage and current commands were provided using a Multiclamp 700B amplifier (Molecular Devices) and signals were filtered using the Digidata 1440A series (Molecular Devices). At the end of each experiment, we examined whether a neuron contained dopamine D_2_ receptors by addition of an agonist, quinpirole (3 µM, Sigma Aldrich), which blocked spontaneous firing. Most of the dopaminergic (DA) neurons (except the amygdala projecting neurons) in the VTA are inhibited by quinpirole [29]. Therefore, using this criterion we were probably excluding a sub-population of neurons. We performed patch-clamp recordings in the region near the MT, where I_h_ current strongly predicts DA content of neuron [30], [31]. Therefore, we attempted to break into whole-cell configuration after the long cell-attached recordings to determine the presence of I_h_ current. To accomplish this, the cells were held at −60 mV and −10 mV, 500 ms hyperpolarizing steps were provided from −60 mV up to −150 mV [32]. However, after long cell-attached experiments, we were only successful in breaking into whole-cell and determining I_h_ in a subset of cells and the magnitude of I_h_ was variable. Thus, the presence of I_h_ current could not be used as an indicator for DA neurons, and instead we examined quinpirole inhibition of firing in the cell-attached mode. Orexin-A and -B peptides were dissolved in DMSO and added to aCSF to a final concentration of 100 nM. Almorexant was freshly made by initially dissolving in 100% DMSO and then in aCSF to achieve a final concentration of 1 µM. Slices were preincubated in almorexant for at least 60 min prior to recording [33]. All reagents were superfused in the recording chamber. Firing rate of neurons was calculated using Clampfit 9.0 (Molecular Devices) and converted to averages of 1 min intervals using Igor Pro 5 software (WaveMetrics, Inc., Lake Oswego, OR, USA). Baseline firing rate was measured before orexin peptides reached the recording chamber. Percent change from baseline was calculated using GraphPad Prism 5.0 (GraphPad Software, Inc. La Jolla, CA, USA).

### 7. Data Analysis

Statistical analyses were performed using SigmaStat version 3.5 (Systat Software, San Jose, CA, USA). Behavioral data for systemic and intra-VTA studies (active and inactive lever presses, g/kg/30 min intake estimated from responses on the lickometer as described above) were analyzed by repeated measures one-way ANOVA followed by Newman-Keuls post hoc analysis when a significant overall main effect was found (*p*<0.05). Data for the intra-SNr infusions and locomotor experiments were analyzed by Students t-tests. Electrophysiological data were analyzed by paired t-test to compare change in firing rate between baseline and orexin application. We also examined across experiments using a one way ANOVA followed by Newman-Keuls post hoc analysis and significance assumed when *p*<0.05. All data are presented as mean ± SEM.

## Results

### Effect of systemic injections of almorexant on 20% ethanol and 5% sucrose self-administration

**Figure 1:**
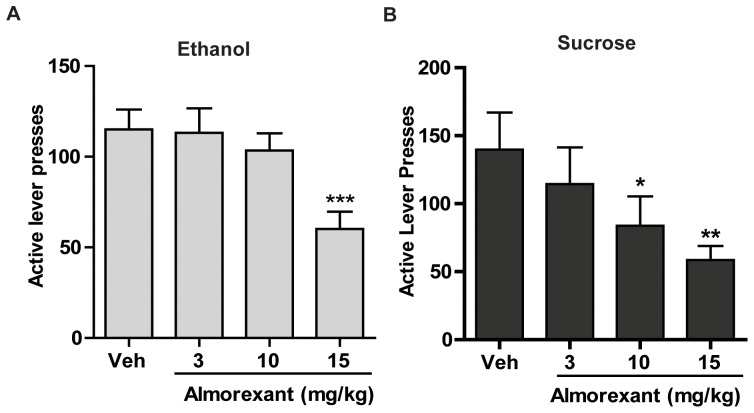
Systemic injections of almorexant reduced ethanol and sucrose self-administration. **A.** After 8 weeks of 20% ethanol self-administration, almorexant significantly attenuated active lever responding for ethanol in Long-Evans rats. **B.** After 8 weeks of 5% sucrose self-administration, almorexant significantly attenuated active lever responding for sucrose in Long-Evans rats. The values are expressed as mean number of active lever presses ± SEM, (repeated measures ANOVA followed by Newman-Keuls post hoc test) ***p<0.001, **p<0.01, *p<0.05 compared to vehicle, n = 14 for ethanol, n = 12 for sucrose.

Our first aim was to test the efficacy of the dual orexin receptor antagonist, almorexant, in reducing ethanol and sucrose self-administration in Long-Evans rats. Previous reports have examined the effects of the Ox-R1 antagonist, SB-334867, on ethanol-seeking behavior [14], [15]. Here, we administered the dual Ox-R1/R2 antagonist almorexant (3, 10, or 15 mg/kg, i.p.) or vehicle 30 min prior to the onset of regular, reinforced self-administration sessions. Almorexant significantly reduced responding on the active lever for ethanol self-administration. A repeated measures one-way ANOVA for ethanol-trained animals revealed a significant effect of almorexant treatment on active lever presses [*F*(3,55) = 16.22, *p*<0.001, Fig. 1A, n = 14], ethanol intake (g/kg) [*F*(3,55) = 20.95, *p*<0.001, Table 1], and reinforcers earned [*F*(3,55) = 20.28, *p*<0.001, Table 1] but no effect on inactive lever presses [*F*(3,55) = 0.38, n.s., Table 1]. Post hoc analysis revealed that the 15 mg/kg dose of almorexant significantly decreased active lever responding (*p*<0.001, Fig. 1A), ethanol intake (*p*<0.001, Table 1), and reinforcers earned (*p*<0.001, Table 1) when compared to vehicle.

**Table 1:** Number of inactive lever presses, amount of ethanol consumed (g/kg), and reinforcers earned following systemic almorexant treatment in animals trained to self-administer either ethanol or sucrose.

Almorexant (mg/kg)	Systemic administration					
	Ethanol self-administration (N = 14)			Sucrose self-administration (N = 12)		
	Inactive lever	g/kg	reinforcers	Inactive lever	g/kg	reinforcers
0	0.36±0.17	1.01±0.11	32.79±3.50	0.42±0.26	0.42±0.11	38.08±8.08
3	0.43±0.17	0.95±0.10	30.71±3.35	0.50±0.42	0.36±0.09	30.17±7.03
10	0.43±0.20	0.90±0.11	29.14±3.60	0.33±0.19	0.30±0.08	22.92±6.47*
15	0.21±0.11	0.50±0.08***	16.00±2.61***	1.25±1.00	0.19±0.04*	16.17±3.03**

Almorexant was administered systemically 30 min before the session. Mean ± SEM is reported.

*** *p*<0.001,

** *p*<0.01,

* *p*<0.05 compared to vehicle.

Similar to the effects on ethanol, almorexant reduced responding on the active lever for sucrose self-administration. Repeated measures one-way ANOVA for sucrose responding animals revealed a significant effect of almorexant treatment on active lever presses [*F*(3,47) = 6.13, *p*<0.01, Fig. 1B, n = 12], sucrose intake (g/kg) [*F*(3,47) = 4.23, *p*<0.05, Table 1], and reinforcers earned [*F*(3,47) = 6.46, *p* = 0.001, Table 1], but no effect on inactive lever presses was observed [*F*(3,47) = 0.62, n.s., Table 1]. Post hoc analysis of the sucrose data revealed a significant decrease in active lever responding (10 mg/kg, *p*<0.05; 15 mg/kg, *p*<0.01; Fig. 1B), sucrose intake (15 mg/kg, *p*<0.01; Table 1), and reinforcers earned (10 mg/kg, *p*<0.01; 15 mg/kg, *p*<0.001; Table 1) following pretreatment with almorexant when compared to vehicle. One animal from the ethanol group and two animals from the sucrose group were excluded due to failure to meet acquisition criteria. Unlike our previous report where systemic injections of SB-334867 did not alter sucrose self-administration [14], we demonstrate that almorexant pretreatment attenuates sucrose self-administration.

### Effect of systemic injections of almorexant on general locomotor behavior

Orexin receptors have been linked to sleep-wake cycles. In fact, almorexant was in Phase III clinical trials for treating sleep disorders by promoting sleep [25]. Thus, we performed locomotor activity experiments to examine whether systemic administration of almorexant at the doses used in the present study might inhibit ethanol and sucrose self-administration through general effects on locomotor behavior rather than its role in the reward circuitry. Following habituation to the locomotor activity boxes, almorexant (15 mg/kg) or vehicle were administered to naïve Long-Evans rats (n = 12) and the ambulatory distance and stereotypic counts were recorded for 60 min. Animals were exposed to almorexant for the same duration (60 min) in the locomotor study and in the systemic self-administration studies. Almorexant induced no significant effects on locomotor activity compared to vehicle (*p* = 0.83 and *p* = 0.98, n.s., for ambulatory distance and stereotypic counts, respectively, Table 2). A previous study that used a higher dose of almorexant (30, 100 and 300 mg/kg administered orally) also did not observe reduced motor performances in rats [34]. In addition, in sleep studies 10 mg/kg almorexant did not decrease alertness, while 30 mg/kg did [35]. The highest dose of systemic almorexant administered in the present study was 15 mg/kg.

**Table 2:** Locomotor responses following systemic almorexant treatment in naïve animals.

Almorexant (mg/kg)	Locomotor Behavior	
	Ambulatory Distance (cm)	Stereotypic Counts
0	930.37±270.70	3273.50±393.46
15	997.57±162.48	3260.17±305.07

Following habituation to the locomotor chambers, almorexant was administered systemically and behavior was monitored for 60 min, n = 12. Mean ± SEM is reported.

### Effect of Microinfusions of Almorexant into the VTA on 20% Ethanol and 5% Sucrose Self-Administration

**Figure 2:**
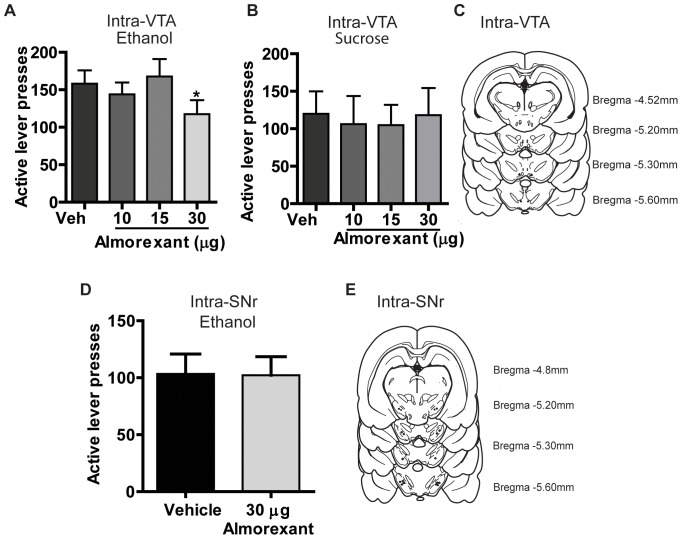
Intra-VTA, but not intra-SNr, infusions reduce ethanol self-administration, but have no effect on sucrose self-administration. **A.** Infusions of almorexant into the VTA significantly reduced active lever responding for 20% ethanol but (**B.**) had no effect on 5% sucrose responding. **C.** Schematic representation of the VTA cannula placements. **D.** Infusions of almorexant into the SNr had no effect on active lever responding for 20% ethanol. **E.** Schematic representation of the SNr cannula placements. Values are expressed as mean active lever presses ± SEM (repeated measures ANOVA followed by Newman Keuls post hoc testing,* *p*<0.05 compared to vehicle for VTA groups and paired t-test for SNr data), n = 12 for VTA ethanol, n = 8 for VTA sucrose and n = 9 for the SNr ethanol.

Our next aim was to identify the importance of the VTA in mediating the effects of almorexant on ethanol and sucrose-seeking behaviors. Previous reports demonstrate that the VTA can play an important role in ethanol-seeking behavior [36], [37], [38], [39], but the role of VTA orexin in ethanol self-administration has not been investigated. Therefore, to examine the effects of intra-VTA almorexant on responding for ethanol or sucrose, we infused almorexant (10, 15 or 30 µg per side) or vehicle 10 min prior to the onset of self-administration sessions. We found that intra-VTA microinfusion of almorexant decreased ethanol responding. Repeated measures one-way ANOVA for ethanol-trained animals revealed a significant effect of almorexant treatment on active lever presses [*F*(3,47) = 12.48, *p*<0.01, Fig. 2A, n = 12], ethanol intake (g/kg) [*F*(3,47) = 24.62, *p*<0.001, Table 3], and reinforcers earned [*F*(3,47) = 25.29, *p*<0.001, Table 3], but no effect on inactive lever presses was observed [*F*(3,47) = 0.85, n.s., Table 3]. Post hoc analysis revealed a significant decrease in active lever responding (*p*<0.05, Fig. 2A), ethanol intake (*p*<0.001, Table 3), and reinforcers earned (*p*<0.001, Table 3) following pretreatment with the 30 µg dose when compared to vehicle. However, intra-VTA microinfusions of almorexant had no effect on sucrose self-administration. Repeated measures one-way ANOVA for sucrose responding animals revealed no significant effect of almorexant treatment on active lever presses [*F*(3,31) = 0.54, n.s., Fig. 2B, n = 8], no effect on sucrose intake (g/kg) [*F*(3,31) = 0.10, n.s., Table 3], no effect on reinforcers earned [*F*(3,31) = 0.11, n.s., Table 3] and no effect on inactive lever presses [*F*(3,31) = 0.41, n.s., Table 3]. One animal from each group was excluded because of failure to complete the dose response due to problems with cannula patency, one animal from the sucrose group was excluded due to failure to meet acquisition criteria, and one animal from the ethanol group was excluded due to incorrect cannula placement.

**Table 3:** Number of inactive lever presses, amount of intake (g/kg), and reinforcers earned following intra-VTA almorexant treatment.

Almorexant (µg)	Intra-VTA infusions					
	Ethanol self-administration (N = 12)			Sucrose self-administration (N = 8)		
	Inactive lever	g/kg	Reinforcers	Inactive lever	g/kg	Reinforcers
0	1.77±0.51	1.43±0.16	41.63±4.45	1.38±0.60	0.34±0.09	33.63±9.20
10	1.20±0.51	1.33±0.14	38.33±3.96	0.88±0.74	0.32±0.11	31.75±11.16
15	0.73±0.21	1.44±0.18	41.67±5.23	0.75±0.41	0.34±0.10	33.13±9.65
30	1.93±1.18	1.08±0.16***	31.42±4.66***	0.75±0.31	0.34±0.12	33.38±11.39

Almorexant was administered intra-VTA ten min prior to the session. Mean ± SEM is reported.

*** *p*<0.001 compared to vehicle.

### Effect of Microinfusions of Almorexant into the SNr on 20% Ethanol Self-Administration

We next examined whether the effects of almorexant on ethanol-self administration in the VTA were specific to this brain locus by administering the compound into the nearby SNr as a site-specific anatomical control. Electrophysiological studies indicate that orexin-A excites SNr GABAergic neurons in rats [40]. We chose SNr as the site specific diffusion control due to its promixity to the VTA and previously described orexinergic action. We hypothesized that SNr orexin receptors are not involved in mediating ethanol self-administration. We found that intra-SNr microinfusion of almorexant (30 µg per side) or vehicle 10 min prior to the onset of the self-administration sessions had no effect on ethanol responding. A paired t-test revealed no significant effect of almorexant treatment on active lever presses (*p* = 0.94, Fig. 2D, n = 9), ethanol intake (g/kg) (*p* = 0.99, Table 4), reinforcers earned (*p* = 0.97, Table 4) and inactive lever presses (*p* = 0.24, Table 4). These results indicate that the VTA is a site of action of the almorexant-induced reduction in ethanol self-administration. Taken together, our data demonstrate that systemic administration of almorexant reduced operant self-administration of ethanol and sucrose, without affecting motor performance, and the effect on ethanol-seeking is mediated (at least in part) by the VTA.

**Table 4:** Number of inactive lever presses, amount of intake (g/kg), and reinforcers earned following intra-SNr almorexant treatment.

Almorexant (µg)	Intra-SNr infusions		
	Ethanol self-administration (N = 9)		
	Inactive lever	g/kg	Reinforcers
0	1.11±0.69	0.95±0.19	27.67±4.92
30	0.33±0.18	0.95±0.18	27.56±4.71

Almorexant was administered intra-SNr ten min prior to the session. Mean ± SEM is reported.

### Almorexant inhibits intracellular calcium signaling at both orexin-R_1_ and orexin-R_2_ receptors

**Figure 3:**
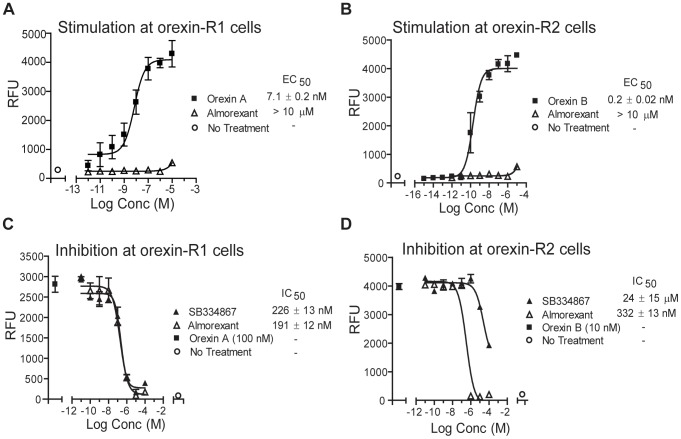
Almorexant inhibits intracellular calcium release in HEK-293 cells expressing either Ox-R1 or Ox-R2. **A.** Activation of intracellular calcium release in HEK-293 cells expressing human Ox-R1 by orexin-A (1 pM-10 µM) (closed squares). Almorexant alone did not stimulate intracellular calcium release in Ox-R1 expressing cells (open triangles). **B.** Activation of intracellular calcium release in HEK-293 cells expressing human Ox-R2 by orexin-B (1 fM- 10 µM, closed squares). Almorexant alone did not stimulate intracellular calcium release in Ox-R2 expressing cells (open triangles). **C.** Inhibition of orexin-A (100 nM, closed squares) induced stimulation calcium release in Ox-R1 by SB-334867 (closed triangles) or almorexant (open triangles) (10 pM-100 µM). **D.** Inhibition of orexin-B (10 nM; closed square) induced stimulation of calcium release in Ox-R2 by SB-334867 (closed triangles) or almorexant (10 pM-100 µM, open triangles). Results are expressed as mean ± SEM relative fluorescence units (RFU) calculated as agonist-induced maximum calcium peak/cell number ×1000.

Almorexant manufactured by Actelion Pharmaceuticals Ltd. is not commercially available. Thus, the almorexant hydrochloride used in the present study was synthesized by Suzhou Rovathin (Jiangsu, China). Therefore, we wanted to confirm the efficacy of this synthesized compound by replicating previous studies that found that almorexant blocks intracellular calcium signaling at Ox-R1 and Ox-R2 receptors [33], [35]. Using the fluorescence based calcium mobilization assay in Human Embryonic Kidney Cells (HEK Cells), full dose-response curves were generated for orexin-A at Ox-R1 (EC_50_ = 7.1±2.0 nM; E_max_ = 4001±106 RFU, mean ± SEM; Fig. 3A) and orexin-B at Ox-R2 (EC_50_ = 0.2±0.02 nM; E_max_ = 3960±35.7 RFU; Fig. 3B). Almorexant alone did not induce intracellular calcium release at either Ox-R1 (EC_50_>10 µM; E_max_ = 318±13.2, *p*<0.01, compared to orexin-A; Fig. 3A) or Ox-R2 (EC_50_>10 µM; E_max_ = 376±52.8 RFU, *p*<0.001, compared to orexin-B; Fig. 3B). We then examined inhibition of orexin-A induced changes in intracellular calcium fluorescence through orexin-R_1_ using the compounds, SB-334867 and almorexant. Orexin-A (100 nM) induced intracellular calcium release was dose-dependently inhibited by SB-334867 (IC_50_ = 226±13 nM) and by almorexant (IC_50_ = 191±12 nM; Fig. 3C). Therefore, both antagonists blocked orexin-A induced calcium changes at the Ox-R1. Next, we investigated whether orexin-B induced changes in intracellular calcium fluorescence at the Ox-R2 receptor were blocked by almorexant. Orexin-B (10 nM) induced changes were dose-dependently inhibited by almorexant (IC_50_ = 332±13 nM; Fig. 3D). However, SB-334867 demonstrated only a partial inhibition of orexin-B induced changes at Ox-R2 (IC_50_ = 24±15 µM; Fig. 3D). Taken together, and similar to the results observed in previous studies [33], [35], the intracellular calcium signaling assay confirms that the almorexant used in this study is a dual orexin R_1_ and R_2_ receptor antagonist, whereas SB-334867 is a more potent antagonist at Ox-R1 compared to Ox-R2.

### Orexin peptides induce increases in spontaneous firing in neurons in the VTA

Previous electrophysiological studies have documented almorexant inhibition of the effect of orexins in Sprague-Dawley and Wistar rats [33], [41], [42]. To further verify the functionality of the almorexant used in this study, we performed *in vitro* electrophysiology experiments. We first tested the ability of orexins to induce firing in the VTA slices obtained from Long-Evans rats. We performed extracellular cell-attached recordings on VTA neurons. Recordings were performed on cells in the region very close to the MT, where most neurons are identified as dopaminergic (DA) by the presence of an I_h_ current and Tyrosine Hydroxylase enzyme (TH) [32], [43]. However, we could not reliably break into whole-cell configuration to determine the presence of I_h_ because this was performed at the end of long cell-attached experiments. Instead, we used the D_2_ receptor agonist, quinpirole, induced inhibition of firing as an indicator of presumed DA neurons [29].

**Figure 4:**
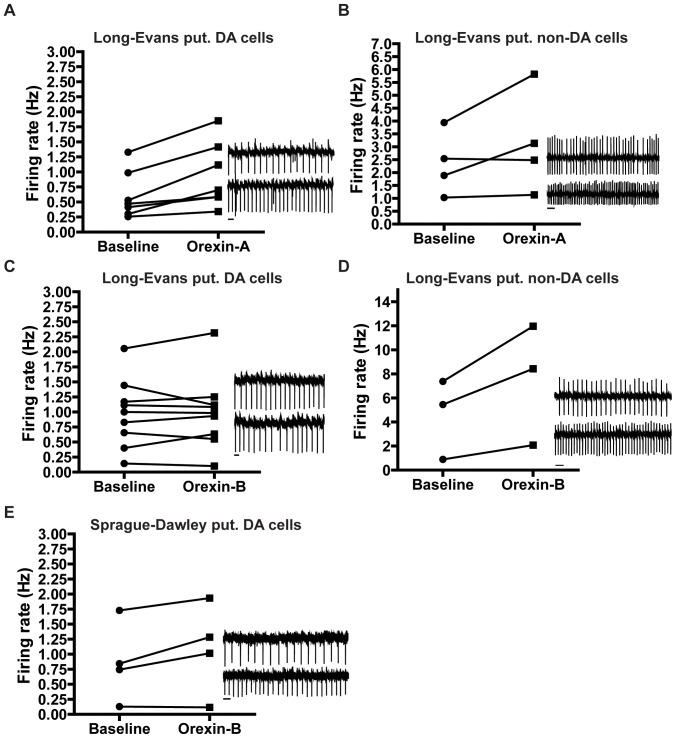
Orexins enhance firing rate in VTA neurons. **A–E.** Graphs of firing rate responses of individual cells to application of orexin peptides. One minute of baseline and the last minute of orexin application are plotted. Sample cell-attached recordings next to the plots demonstrate effect of orexin-A or -B on firing rate. Baseline before addition of orexin peptide (top trace) and last minute after addition of orexin (bottom trace) are shown, scale bar denotes 10 s. **A.** Responses of Long-Evans putative DA neurons to orexin-A, where 6 out of 7 neurons increased in firing with orexin-A. **B.** Responses of Long-Evans putative non DA neurons, where 2 out of 4 neurons increased in firing with orexin-A. **C.** Responses of Long-Evans putative DA neurons to orexin-B, where 2 out of 9 neurons responded to orexin-B. **D.** Responses of Long-Evans putative non-DA neurons, where all cells increased in firing rate upon orexin-B application. **E.** Responses of Sprague-Dawley putative DA neurons, where 2 out of 4 significantly increased in firing with orexin-B. Paired t-test was used to assume significance.

From a total of 11 neurons tested, 8 neurons showed an increase in firing upon bath application of orexin-A (100 nM). Of the seven putative DA neurons tested, 6 neurons increased firing upon application of orexin-A (paired t-test, p<0.01, Fig. 4A). Orexin-A also induced firing rate in 2 out of 4 putative non-DA neurons (paired t-test, p<0.01, Fig. 4B); in these neurons, quinpirole did not inhibit firing. Compared to orexin-A, superfusion of orexin-B (100 nM) modestly increased firing in 2 of 9 putative DA neurons (Fig. 4C). However in putative non-DA neurons, orexin-B application increased in firing rate in all three neurons tested (paired t-test, p<0.01, n = 3, Fig. 4D). The increase in firing with orexin-A (100 nM) in DA neurons was similar to responses observed in Wistar rats [41]. However, our weak response in putative DA neurons to orexin-B contrasts with the enhanced firing and potentiated glutamatergic transmission in VTA DA neurons from Wistar and Sprague-Dawley rats, respectively [41], [42]. We, therefore, hypothesized that the weak response for orexin-B in our study was due to strain differences between Long-Evans and Sprague-Dawley rats. Application of orexin-B in VTA slices from Sprague-Dawley rats demonstrated a significant increase in firing in 2 of the 4 putative DA neurons tested (p<0.01, Fig. 4E). One putative DA neuron showed a non-significant increase in firing (Fig. 4E) and 1 of 2 putative non-DA neurons showed a significant increase (data not shown).

### Almorexant blocks orexin-A induced enhancement of firing rate

**Figure 5:**
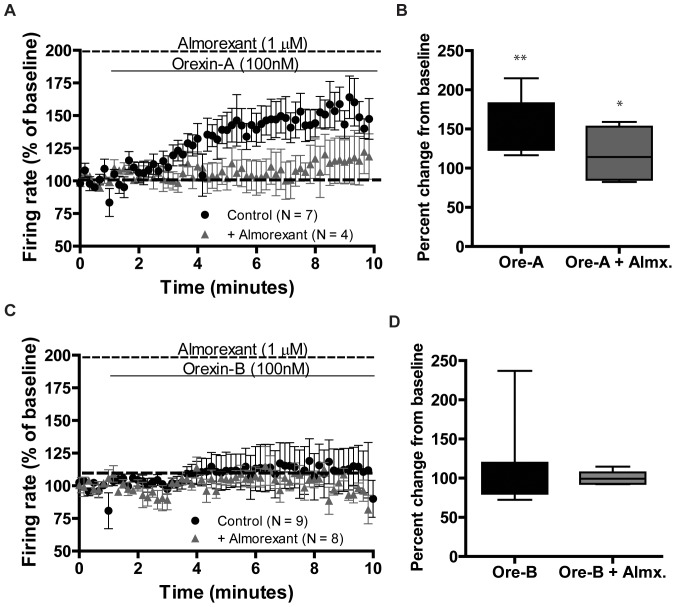
Almorexant blocks orexin-A induced increase in firing rate. **A.** Almorexant (1 µM) blocked orexin-A (100 nM) induced enhancement of firing rate in Long-Evans VTA neurons, n = 4. **B.** Percent change in firing rate from baseline, denoted as 100%, in the presence (grey bar, ore-A+Almx.) or absence of almorexant (black bar, ore-A) upon application of orexin-A. One-way ANOVA followed by Newman-Keuls post hoc test comparing the baseline (1 min) and last one min of orexin-A application (** *p*<0.01, plotted from fig. 5A, control). *p<0.05 denotes comparison between last min of orexin-A only (control, Fig. 5A) and orexin-A+Almorexant (+Almorexant, Fig. 5A). **C.** No change in firing rate upon orexin-B (100 nM) application was observed in presence of almorexant, n = 8. **A & C.** Each point represents mean ± SEM over a period of 10 seconds. **D.** Percent change in firing rate from baseline, denoted as 100%, in the presence (grey bar, ore-B+Almx.) or absence of almorexant (black bar, ore-B) upon application of orexin-B.

We next tested the efficacy of almorexant to block orexin-induced increase in firing in VTA neurons from Long-Evans rats. Almorexant prevented the increase in firing with orexin-A. One-way ANOVA comparing orexin-A induced increase in firing in the presence and absence of almorexant revealed an overall main effect of drug treatment [*F*(3,23) = 5.337, *p* = 0.007] (Fig. 5A & B). Post hoc analysis revealed that almorexant significantly blocked the orexin-A-induced increase in firing (Fig. 5B, *p*<0.05). Although, we observed a weak effect of orexin-B in putative DA neurons (Fig. 5C & D), no significant effect was observed after almorexant preincubation [*F*(3,29) = 0.0236, *p* = 0.99] (Fig. 5C & D). Taken together, our results indicate that the almorexant used in this study is able to block the orexin-A induced increase in firing in VTA neurons from Long-Evans rats.

## Discussion

Recent advancements in understanding the orexin/hypocretin system have provided insights into its role in drug abuse [11], [12], [13], [14], [17] specifically in AUDs. The major aim of this study was to characterize the behavioral effects of the dual orexin receptor antagonist, almorexant, in the context of developing new pharmacotherapies for the treatment of AUDs and maladaptive food intake. Here, using an operant self-administration paradigm, we report that systemic injections of almorexant reduced both ethanol and sucrose self-administration. Further, we use intracranial infusions to demonstrate that VTA orexin receptors support ethanol, but not sucrose, self-administration, suggesting that orexin promotes ethanol and sucrose self-administration through different mechanisms within the brain. We also verify using an *in vitro* calcium fluorescence assay that the almorexant used in this study, was effective in blocking orexin induced activation of both Ox-R1 and Ox-R2 receptors. Lastly, using *in vitro* electrophysiological techniques, we confirmed almorexant was able to inhibit orexin-A induced increase in firing in VTA neurons of Long-Evans rats.

Previous studies have demonstrated that the Ox-R1 antagonist, SB-334867, reduces operant self-administration of ethanol in Long-Evans rats [14] and prevents cue-induced reinstatement of ethanol-seeking in alcohol preferring rats [15]. In addition, a recent report [18] demonstrated that systemic administration of Ox-R2 receptor antagonist, JNJ-10397049, also attenuated ethanol self-administration. To date, the role of these receptors in reward-seeking behaviors has been unclear due to the lack of publicly available Ox-R2 antagonists. Here, we demonstrate that the dual R1/R2 blocker almorexant was effective in reducing ethanol self-administration in Long-Evans rats. Together, these findings suggest that modulating activity at either orexin receptor subtypes may be beneficial for the treatment of AUDs, and support our findings that almorexant may represent an effective treatment for AUDs.

In the present study, almorexant also significantly attenuated sucrose self-administration. Orexin peptides have been shown to stimulate feeding behaviors [2]. ICV injections of orexin-A stimulate feeding and delay behavioral satiety [44], whereas an anti-orexin A antibody suppresses food intake in rats [8]. Additionally, intraperitoneal injections of SB-334867 have been shown to reduce food intake [45]. Taken together, these findings suggest a strong correlation between orexin and feeding behavior. Previous studies using SB-334867 have shown variable effects on sucrose-seeking. Earlier work from our laboratory using Long-Evans rats demonstrated that SB-334867 (20 mg/kg) did not reduce sucrose self-administration [14], whereas in alcohol preferring rats, SB-334867 (5 mg/kg) reduces sucrose-seeking under a fixed-ratio schedule of reinforcement, but not under progressive ratio of reinforcement [16]. In fact, there are mixed results regarding the efficacy of SB-334867 in reducing responding for natural rewards, most of which have been attributed to the dose administered, the reinforcement schedule and the reinforcer itself (natural rewards, such as chow and sucrose, compared to novel rewards, such as alcohol, high-fat food and cocaine) [16], [46]. Since almorexant is a dual receptor antagonist, we are unable to dissociate the effects between R1 and R2 receptors. Currently, there is no current evidence for the involvement of Ox-R2 in sucrose-seeking, investigations using selective Ox-R2 antagonists are required to confirm the role of these receptors in sucrose-seeking behavior.

The VTA has been shown to have a robust expression of Ox-R1 and Ox-R2, and orexinergic terminals synapse onto presumed dopaminergic and GABAergic neurons [11], [47], [48]. Orexin-A induces potentiation of NMDAR-mediated neurotransmission via a PLC/PKC-dependent insertion of NMDARs in VTA DA terminals and induces behavioral sensitization to cocaine [12]. As shown here and elsewhere [41], orexin-A also increases firing in VTA neurons. Similar to observations in Wistar rats [33], we demonstrate that almorexant blocks orexin-A induced increase in firing rate in Long-Evans rats. In our experiments, orexin-B seldom increased firing rate in putative DA neurons, but did increase firing in putative non-DA neurons from Long-Evans rats. In contrast, in Sprague-Dawley rats, we observed a greater proportion of putative DA cells that responded to orexin-B. It is possible that there is lower expression of orexin-B specific Ox-R2s in VTA DA neurons of Long-Evans rats. Ox-R1 receptor mRNA, but not Ox-R2, was found in a majority of isolated VTA neurons from Wistar rats [41]. Since several strong differences have been observed across species and rat strains, for instance in the dopamine system [49], [50], we speculate that distribution of orexin receptor expression could also be strain specific.

The role of the VTA in drug-seeking behavior is well documented [51]. Intra-VTA infusions of orexin increase dopamine release into the prefrontal cortex, but not in the nucleus accumbens [52]. *In vitro* orexin application in the VTA augments DA neuron responses to mPFC stimulation [53]. Our results that orexin-A augments firing rate in neurons hyperpolarized by D2 receptor agonist suggest that we might be targeting the PFC projecting DA population in addition to NAc projecting neurons [29]. Intra-VTA, but not paraventricular thalamus, infusions of SB-334867 attenuate cue-induced reinstatement of cocaine-seeking [54]. Our observation that intra-VTA infusions of almorexant attenuated ethanol self-administration provides support for a role for orexin receptors in the VTA in drug-seeking behaviors. The lack of effect following intra-SNr infusions of almorexant, despite the presence of orexin immunoreactivity in that region [40], confirms that the effect on ethanol self-administration was specific to the VTA.

Interestingly, the effect of almorexant in the VTA seems to be specific for ethanol. In contrast to the reduced sucrose responding following systemic administration of almorexant, intra-VTA administration of almorexant had no effect on self-administration of sucrose. There are many plausible explanations for a lack of an effect in the VTA, including (1) that distinct brain circuits modulate sucrose and ethanol-seeking behaviors and (2) that the orexin receptor subtypes play disparate roles in ethanol and sucrose-seeking behaviors. Several hypothalamic circuits control food consumption and have overlapping connections with the mesolimbic reward circuitry [55]. Orexin is one of the neurochemicals that is capable of regulating both food and drug intake [56]. Since orexinergic fibers innervate various brain regions, it is possible the sucrose-seeking behavior is mediated in regions other than the VTA. In addition, previous work has established a dichotomy in orexinergic function, where orexins expressed in the LH activate reward-seeking behavior, while orexins expressed in the perifornical nucleus and dorsomedial hypothalamic nucleus activate arousal- and stress-related behaviors [57]. Similarly, there is a differential distribution of Ox-R1 and Ox-R2 receptors in the brain, which are also coupled to different sub-classes of G proteins [47], [58], [59], [60]. Further studies will be required to understand the differences in the orexin system that contribute to differential regulation of ethanol and sucrose intake.

The Ox-R1 specific antagonist, SB-334867, has been the most commonly used orexin receptor antagonist. However, there are problems associated with the poor solubility and specificity of this drug, which perhaps contributes to the mixed results in previous studies. Due to the lack of Ox-R2 or dual receptor antagonists, most of the results obtained from SB-334867 have focused attention towards the Ox-R1 as playing a crucial role in reward-seeking behavior. With the recent availability of the selective Ox-R2 antagonists, the role of these receptors will now become evident. Dual orexin receptor antagonists, like almorexant, provide advantages in pharmacotherapy, since they target both the receptors and are effective in reducing the effects caused by both of them. Our results provide the first preclinical evidence that lower doses of dual orexin receptor antagonists, than used in sleep studies, are effective in reducing ethanol- and sucrose-self-administration in animal models of drinking. The orexin system is a novel target for the treatment of AUDs and regulating excessive food consumption, and dual orexin receptor antagonists like almorexant could serve as novel therapeutic interventions.
